# Different LFP frequency bands convey complementary information about the BOLD signal

**DOI:** 10.1186/1471-2202-12-S1-P204

**Published:** 2011-07-18

**Authors:** Cesare Magri, Ulrich Schridde, Stefano Panzeri, Yusuke Murayama, Nikos K Logothetis

**Affiliations:** 1 Max Planck Institute for Biological Cybernetics, 38 Spemanstrasse, 72076 Tübingen, Germany; 2 Imaging Science and Biomedical Engineering University of Manchester, Manchester, UK; 3 Italian Institute of Technology, Department of Robotics, Brain and Cognitive Sciences, Genova, Italy

## 

Blood-oxygen-level-dependent (BOLD) functional magnetic resonance imaging (fMRI) is the most widely used noninvasive imaging technique for investigating brain activity. However, the BOLD signal is only indirectly coupled to the underlying neural activity and the relationship between the two signals is not fully understood [1]. Recordings in anaesthetized and awake monkeys have shown that hemodynamic responses are strongly related to local field potentials (LFPs) [2,3]. LFPs are thought to represent the input and intracortical processing in a cortical area and are usually separated into different frequency bands that reflect different neural processes [4]. Previous studies have shown that different LFP bands correlate differently with the BOLD signal [3,5,6]. However little is known about which property of the BOLD signal is reflected by each band and whether different bands convey different information about the BOLD signal. To address this question we performed simultaneous recordings of neural activity and BOLD fMRI in early visual areas V1 and V2 in 4 anesthetized monkeys. All measurements were performed with the monkeys sitting in complete darkness while no stimulus was being presented. We computed mutual information between LFP power and BOLD fMRI to determine which frequencies in the LFPs were most informative about the BOLD signal. We found three highly informative bands, namely the alpha band [8-12Hz], the gamma band [40-100Hz] and the [18-35 Hz] “nMod” band that was previously found to be unrelated to visual stimuli and was thus suggested to primarily reflect neuromodulatory input [4]. We found that gamma power was the most informative about BOLD fMRI and reflected well changes in the amplitude of the BOLD signal. In particular, an increase in gamma power above its median value was followed, on average, by an increase in BOLD signal, and the BOLD signal decreased, instead, following a decrease in gamma power below its median. Moreover, we found that gamma and nMod power were complementary, i.e. that by combining nMod power together with gamma power we could extract 30% more information than could be extracted from gamma power alone. We investigated the origin of this complementarity and we found that the power in the nMod band reflected the timing with which changes in BOLD signal occurred following changes in gamma power. Finally, we found that, as suggested by previous theoretical work [7], an increase in alpha power without a change in total LFP power was followed by a decrease in BOLD signal and vice versa. These results indicate that distinct neural processes are reflected differently in the BOLD signal and that, consequently, it may be possible to retrieve information about the different contributions from the recorded BOLD time course.
